# Supplementary material for: Image processing approaches to enhance perivascular space visibility and quantification using MRI
Source: Sci Rep. 2019 Aug 26;9:12351. doi: 10.1038/s41598-019-48910-x (PMC6710285; doi:10.1038/s41598-019-48910-x)
Supplement: Supplementary file 2 — Supplementary figures [file 41598_2019_48910_MOESM2_ESM.docx]

# Image processing approaches to enhance perivascular space visibility and quantification using MRI

**Authors:**
Farshid Sepehrband, Ph.D.^1^*, Giuseppe Barisano, M.D.^1,2^, Nasim Sheikh-Bahaei, M.D., Ph.D.^1,3^, Ryan P Cabeen, Ph.D.^1^, Jeiran Choupan, Ph.D.^1,4^, Meng Law, M.D.^1,5^, and Arthur W. Toga, Ph.D.^1^

**Affiliations:***1. Laboratory of Neuro Imaging, Stevens Neuroimaging and Informatics Institute, Keck School of Medicine, University of Southern California, Los Angeles, CA, USA*

*2. Neuroscience graduate program, University of Southern California, Los Angeles, CA, USA*

*3. Department of Radiology, Keck Hospital of USC, Keck School of Medicine, University of Southern California, Los Angeles, CA, USA*

*4. Department of Psychology, University of Southern California, Los Angeles, CA, USA*

*5. Department of Radiology, Alfred Health, Melbourne, Australia*

*** Correspondence to:**

Farshid Sepehrband, PhD

Laboratory of Neuro Imaging,

[USC Mark and Mary Stevens Neuroimaging and Informatics Institute](http://www.ini.usc.edu/),

Keck School of Medicine of USC, University of Southern California,

Los Angeles, CA, USA

T: (+1) 323-442-7246

E: [farshid.sepehrband@loni.usc.edu](mailto:farshid.sepehrband@loni.usc.edu)

## Supplementary figures


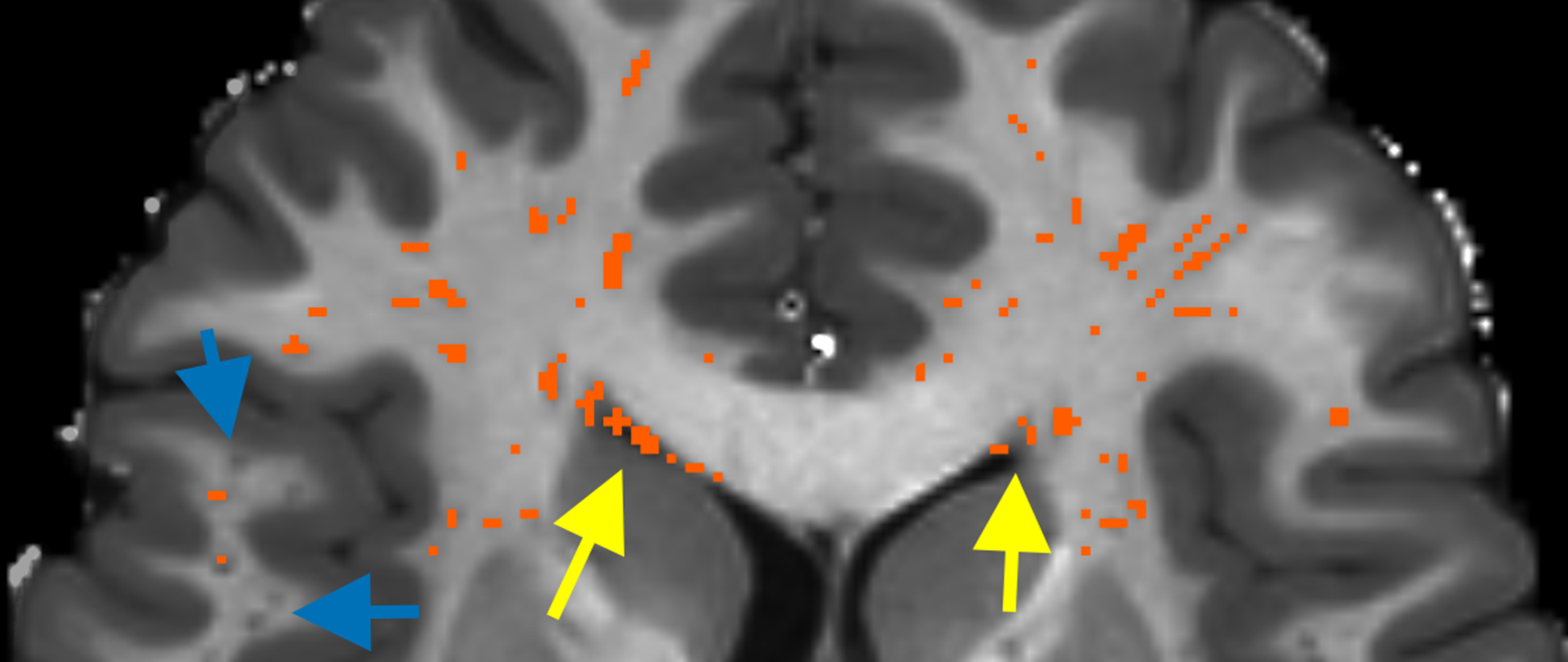


**Supplementary Figure 1**. Examples of negative influence of the imperfect white matter parcellation on perivascular spaces (PVS) segmentation. Yellow arrows show periventricular voxels misclassified as PVS. Blue arrows show voxels with PVS in the superficial white matter which were not included in the white matter parcellation. While false positive voxels in periventricular area were removed by applying a dilated mask of the ventricle, the superficial white matter voxels remained unsolved.


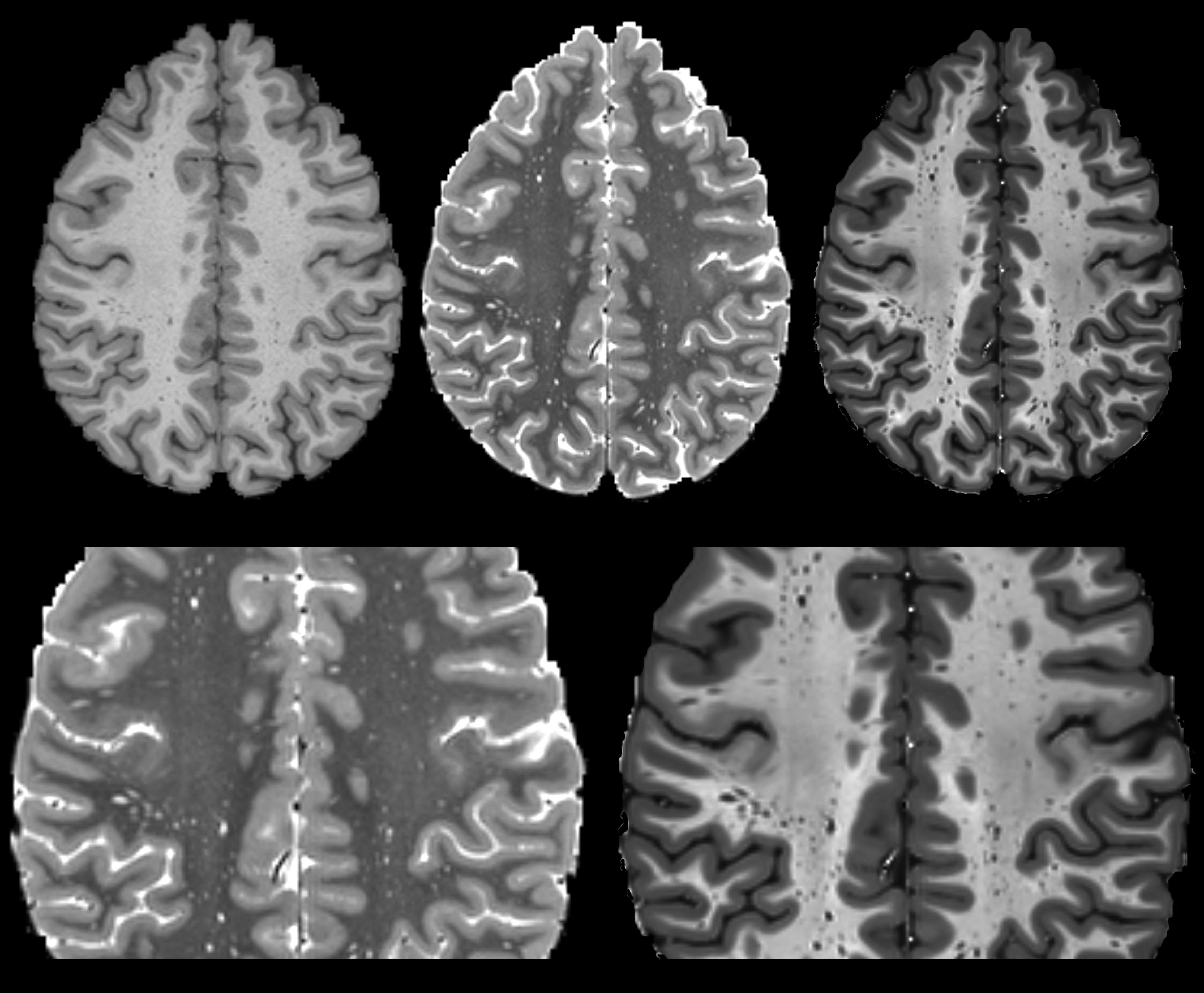


**Supplementary Figure 2**. Comparing Enhanced PVS Contrast (EPC) with T1-weighted and T2-weighted images in subject with large number of small perivascular spaces (PVS) in centrum semi-ovale. Note that EPC aids visual identification of PVS and distinguishing them from image noise.


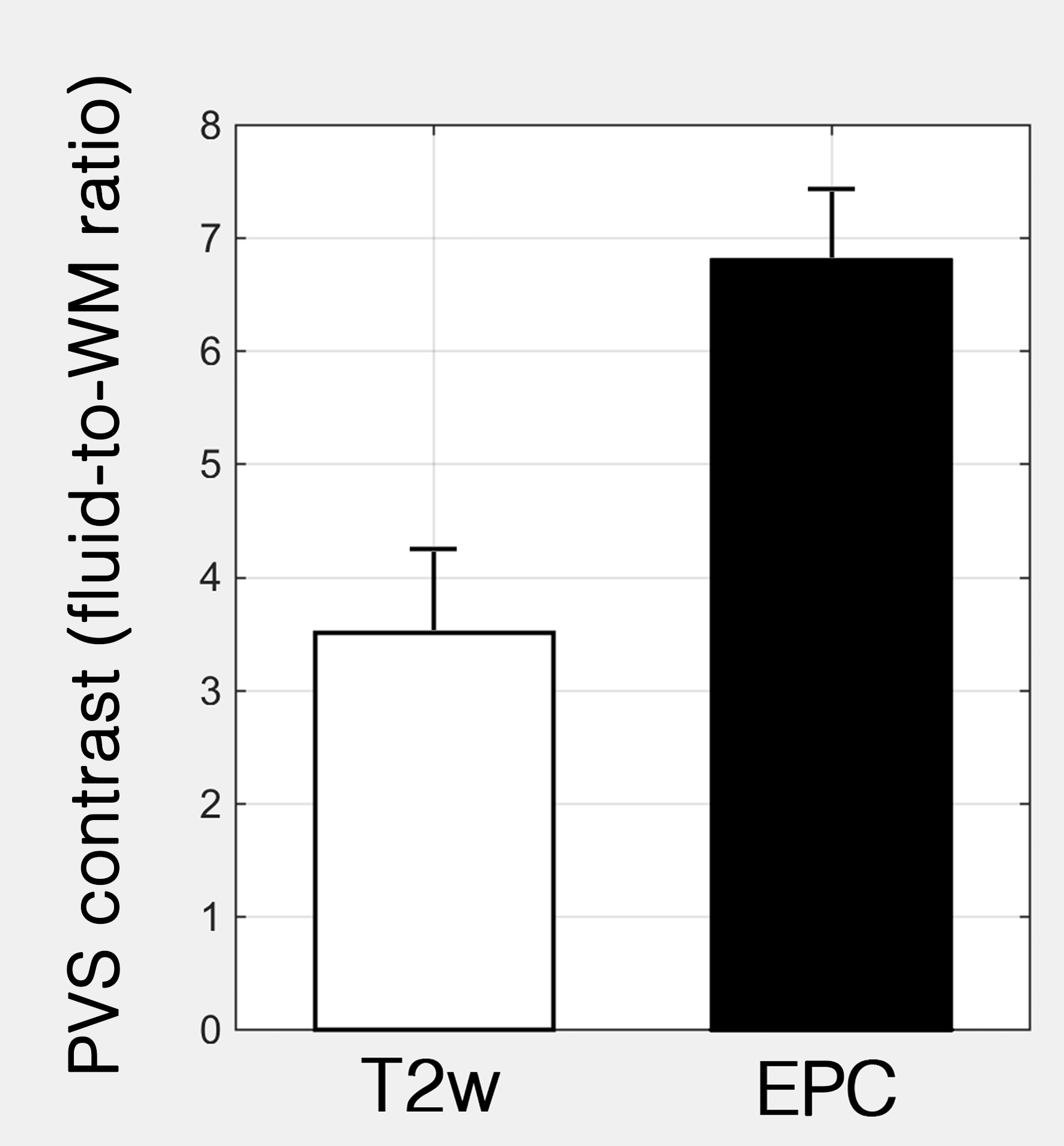


**Supplementary Figure 3**. The contrast ratio between perivascular spaces (PVS) and the adjacent white matter voxels from multiple manually selected regions. The PVS-to-white matter contrast ratio of the EPC was significantly (*p*=0) higher than that derived from T2w images.


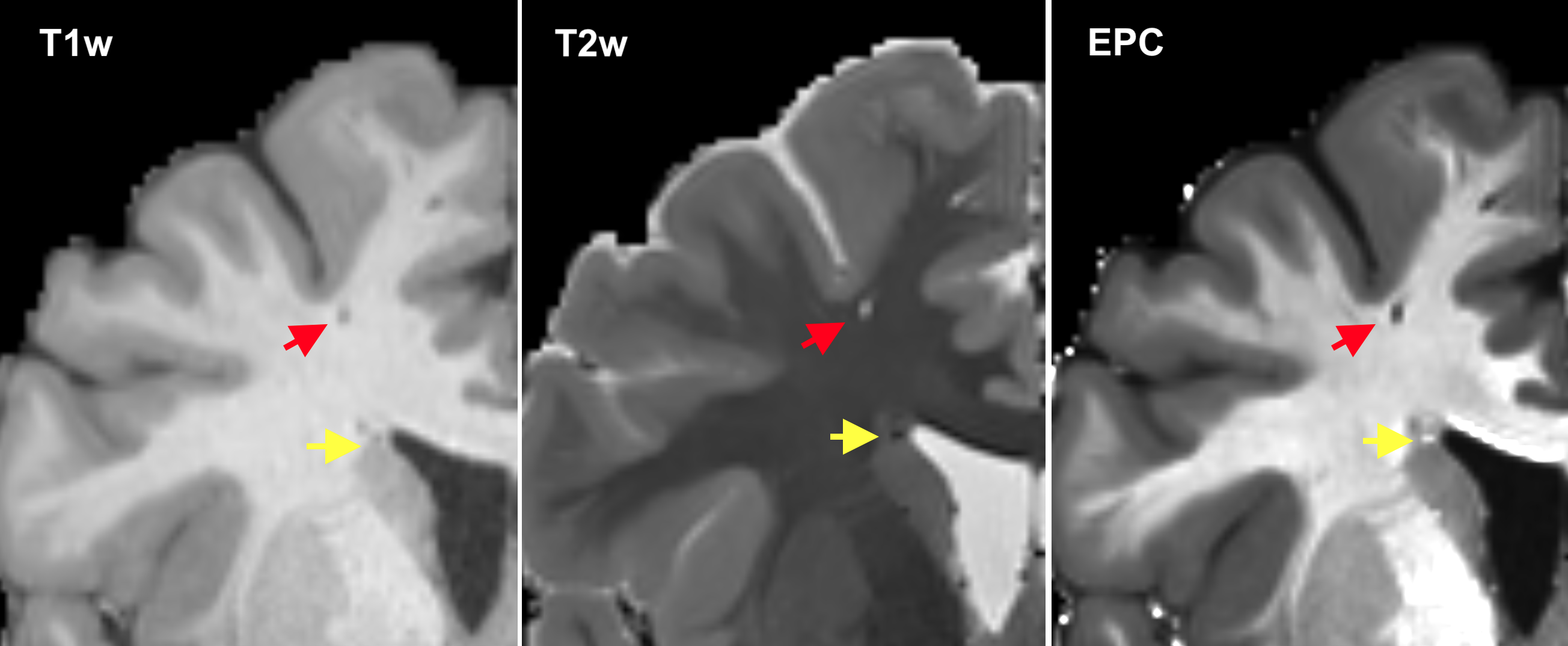


**Supplementary Figure 4**. Different signature of vessels with and without PVS on the EPC. Red arrow shows a vessel with PVS. Yellow arrow shows a vessel with reasonably no surrounding PVS, due to the absence of the PVS-like signal on T2w (i.e. bright signal). The bright and dark voxels indicated by the arrows were followed through multiple slices to ensure they are indeed vessels. Note that differentiation of the PVS would have been incorrect in an automated technique if only T1w was used.
